# Quarantine and the Spread of Epidemic Diseases

**Published:** 1861-04

**Authors:** Gavin Milroy


### Art. IV.—QUARANTINE AND THE SPREAD OF EPIDEMIC DISEASES.

By Gavin Milroy, M.D., F.R.C.P.

Among the means employed in most countries to preserve the public health and to ward off the visitations of pestilential diseases, is that of enforced detention and purification of infected or suspected ships, persons, and goods, before their direct communication with the shore or their passing the frontier is allowed, known as Quarantine. Under this term is also included the practice of isolating a place where a pestilence already exists, by drawing a cordon of troops around it, so as forcibly to cut off all intercourse between it and the surrounding district, in the hope of confining the mischief to the infected spot, and thus stopping its extension.

As thus understood, and as it is generally practised, quarantine can scarcely be regarded as a part of true sanitary or preventive medicine, the high aim and object of which is to prevent the rise and growth of disease by removing the causes which foster their development and give malignancy to their action. It is rather a department of State police, which endeavours, by a sort of mechanical machinery, to keep out the introduction *ab extra* of a dreaded distemper, very much after the same fashion as the Custom-house authorities endeavour to prevent the importation of interdicted foreign goods, or the landing of strangers who have not duly-authorized passports.

Hitherto, with only few and occasional exceptions, the subject has not received from medical men, and others competent to judge, that amount of attention which its great importance, not only in a social and international, as well as a commercial point of view, but also as a question which involves the consideration of some of the most interesting problems of epidemiological research, certainly demands. It would be difficult, indeed, to name any branch of scientific inquiry on which so many frivolous, and even fabulous statements have been received as evidence, and so much loose and inconsequential reasoning has prevailed. The result has been what might be expected—quarantine has ceased to be regarded by men of enlightenment as a safeguard of the public health, and too generally it has become degraded into a mere instrument of mercenary exaction, or perverted to one of vexatious Government interference with the freedom of international intercourse.

That it has failed, and continues to fail, in effecting the object for which it was established, is patent to all. The countries where it is most systematically and stringently exercised are certainly not more free than others from pestilential visitations, and some of them—Portugal and Naples, for example—have suffered more frequently and severely of late years from destructive epidemics than Holland and our own country, where the ordinary restrictions of quarantine are comparatively in abeyance. So notorious, indeed, has been the inefficacy of the system, that some able medical men have recommended its entire abolition, and the discontinuance of all endeavours of the sort, as vain and useless, to keep out any form of disease. It seems wiser, however, rather to seek to reform and remodel a machinery that is capable of being rendered publicly useful, than to do away with it altogether because it has been defective and has been abused.

A rational quarantine should obviously be based upon a knowledge of the ascertained laws which affect and regulate the rise and spread of those diseases which it seeks to exclude, and its regulations and restrictions should as obviously be in consonance with the results of past experience of the measures hitherto employed. The diseases against the importation of which quarantine is, in the present day, mainly directed are plague, cholera, and yellow fever; and it is to them chiefly that the following remarks are meant to apply:—

I. Although we are ignorant of the causes which determine the irregularly periodic outbursts of these diseases in an epidemic or pestilential form, and why they manifest themselves in one year and season, and in one district, island, or country, and not in another under apparently the same condition and circumstances, repeated observation has now satisfactorily demonstrated that they invariably spring up in low-lying damp localities, abounding with vegetable and animal refuse—such as river-banks and deltas, swampy shores or filthy harbours, and where the atmosphere is therefore charged with organic impurities. There appears to be no exception to this law, nor to the like and cognate law that the first cases of the distemper invariably occur in the foul and squalid abodes of the poor. Aerial impurity seems therefore to be as necessary an element or factor in the development of the morbific poison, or germ, as the presence of damp or decaying organic matter is in the development of ordinary mould or mucor. Certain it is that the "materies morbi," as medical writers term it, or the material something which when received into the living body occasions the train of symptoms or phenomena known as plague, or yellow fever, or cholera, ceases to exist, or at least is rendered impotent, in an atmosphere that is kept perfectly pure. The easy means of disinfection are thus always at hand. Even in the height of an epidemic, and in the very midst of an infected district, the neutralizing power of constantly renewed fresh air has often been signally conspicuous, the inmates of clean well-ventilated buildings remaining unscathed while deadly sickness has been raging around.

No fact is better established, and none is more fruitful of important practical instruction, than the fact of the indispensable co-operation of an impure and vitiated atmosphere to the full force and malignancy of all pestilential diseases.

II. An epidemic invasion is not, as is very generally imagined, a sudden or unheralded event. It is usually preceded by various signs or phenomena which the careful observer will seldom, if ever, fail to discover. The meteorological conditions are often irregular and distempered. There is a greater amount of sickliness of different kinds than usual, and the diseases exhibit anomalous and peculiar characters. Generally, however, the prevailing sickness is only a milder and less developed form of the approaching pestilence. Thus, the cholera is usually preceded by epidemic diarrhœa of a choleraic type, or "cholerine," as it has been called by foreign writers; yellow fever by irregular and unusually severe forms of endemic malarial fever, often associated with troublesome bowel disorders—and plague is universally ushered in by typhus fever, which so gradually lapses into the more malignant and dreaded pestilence that it becomes impossible to determine with accuracy when the earliest developed case of the latter took place.

It has been observed that a stranger arriving in a locality where this precursory state of things exist is more liable to sicken than the residents themselves, and that the attack in such circumstances very generally proves fatal, the disease manifesting its malignant type. If this be the first, or one of the first, fatal cases of the epidemic, a suspicion not unnaturally arises in the public mind that the pestilence has been introduced from without, and the suspicion soon gains force, and is readily believed, because every community and country will strive to repudiate the paternity of the evil as a reproach and disgrace, and will seek to father it upon others. The extreme susceptibility of new comers into an infected locality to be attacked by epidemic disease is a fact suggestive of important practical applications in military as well as in civil life.

III. All evidence seems to show that the spread of epidemics is mainly through atmospheric agency, although other and more partial agencies may aid in their dissemination. The diffusion by the atmosphere takes place in a twofold manner. Pestilences are often migratory upon a great scale, travelling on from the country where they sprang up to other and distant countries, and this, too, by successive, although irregular, marches, very much after the similitude of the progression of insect swarms from one region or continent to another.

In former times, the plague, as the "Black Death," steadily advanced from the confines of China—as epidemics of influenza have been known to do in more recent times—across Thibet and Persia to Southern Russia, and thence extended itself over almost every country in Europe. In our own days, the cholera from the delta of the Ganges has been seen to follow nearly the same track, and with like desolation in its course; and within the last few years, the yellow-fever pestilence has exhibited a diffusive energy unknown before, extending in the New World its ravages from the 30th parallel or so of southern latitude to the 40th degree of north latitude, and from the seaboard of Brazil to that of Chili and Panama.

These great migratory movements must be due to an impelling atmospheric power which eludes our knowledge. Is it, however, unreasonable to suppose that, if accurate registers were kept of the exact dates of the development of the disease in different countries, and of other meteorological phenomena at the same time, some connexion might one day be traced between them, and some approach be made to the discovery of a "Law of Epidemics," as there has been of recent years to the discovery of a "Law of Storms?" But all is mystery at present.

When the epidemic poison has reached a district, its mode of diffusion appears generally to be by a larger or smaller number of nearly simultaneous, or quickly successive, scattered spots of infection, or, as it were, of fermentative action; these spots being irregularly detached and separate from each other at first, but gradually enlarging and extending by the development of new and more numerous spots, until at length they may coalesce, and the atmosphere of the entire district is the scene of morbific activity. The very accurate investigation which was instituted by the General Board of Health of all the early cases of the cholera when the pestilence visited London in the autumn of 1848, fairly leads to such an explanation of its mode of spreading in the metropolis; and the table of the dates of its appearance in different parts of England and Wales during 1848 and 1849, given in the Registrar-General's valuable Report on the epidemic, seems to point to the like conclusion.

Besides these modes of general diffusion through the medium of atmospheric agency, a pestilential disease possesses the property of increase and multiplication in the bodies of the sick, and of being, under certain conditions, communicable from the sick to healthy persons around them;—these latter becoming, it may be, under similar circumstances and conditions, the instruments of a wider propagation.

This property is generally known by the term "Contagion,"—a term of very loose and ill-defined import, being indiscriminately applied to the mode of spreading of the itch and of scald head on the one hand, and of the cholera or the plague on the other.

There has been much and vehement controversy among medical writers as to the part which contagion plays in the extension of most pestilential diseases, and as the most extreme opposite opinions have generally been formed and insisted upon by the disputants, the cause of Truth has, as is usual in such cases, but little advanced. To deny unqualifiedly their communicability, and to assert without reserve that they are never contagious, has only exposed the advocates of the doctrine to easy confutation by their opponents, who, on the other hand, have usually adopted the extravagant notion that because under some conditions and in some instances, a disease has manifested a power of transmission from one person to another, not only must it universally and invariably do so, but also that it is in this way alone that it can diffuse and extend itself.

It would be about as reasonable for any one to maintain, that, because trees may be multiplied by slips or cuttings planted in the ground, their propagation is never effected by their seeds being wafted from place to place.

The really important question is to determine the circumstances or conditions in which a disease is apt to manifest a contagious property, and to determine the means, if such exist, by which such property may be neutralized or prevented. Now experience has incontestably shown that, just as the disease never springs up primarily in a place where the atmosphere is fresh and pure, so it cannot continue to exist, far less to thrive, where such an atmosphere prevails. The same aerial condition is necessary for the reproduction as for the original production of the morbific germ.

It is by acting upon and giving effect to this simple rule of sanitary art that Quarantine may be made truly useful, and an instrument of much good. The actually sick, and those in whom the early or premonitory symptoms of the sick are present, should be removed as promptly as possible from the ship on board which they have sickened, and be transferred to clean and airy quarters, either on shore or afloat. Their chances of recovery will thus be greatly promoted, and the risk of the extension of the disease will be reduced to the minimum.

The detention and confinement of the crew and passengers who have been in perfect health during the voyage, and are so upon arrival, merely because they have recently come from a country where an epidemic disease existed when they left, or because a case of sickness may have occurred on board the vessel, and on he suspicion that the disease may be dormant in their systems, or that its germs may be somehow or other clinging to their persons, appear to be unnecessary on the score of the public health, and might be safely discontinued. The practice has been rather based upon theoretical fears than derived from the results of observation and experience.

There is no reliable evidence to show that any pestilential disease was ever introduced into a country by the cargo of a ship, or by the ordinary articles of trade or merchandize; always provided that they were in a fresh, unputrescent condition, and excluding, of course, from the category such articles as the bedding and body clothing of the sick. Among the thousands of men who have been employed, since the beginning of the present century, in the lazarets of Malta, Marseilles, and Genoa, in handling the cotton-bales and other goods on board vessels from Turkey and Egypt, not a single instance of sickness attributable to the occupation has occurred. If this be true in respect to the plague, how much more must it be in respect of yellow fever, or cholera, the contagious property of which is universally admitted to be much less active.

It is to the ship itself far more than to the cargo on board that the attention of quarantine authorities should be directed. As a foul, infected ship may unquestionably be the vehicle of introducing disease, if allowed in this state to lie alongside other vessels, more especially in a crowded port, or close to dwellings on the shore, it is right that the most stringent measures of thorough cleansing and disinfection should be required before she is admitted to free *pratique*.

There is much room for the amendment of the sanitary condition of the merchant shipping of all countries; and just in proportion as this is more attended to, and the sanitary state of seaports is improved, so will the necessity for quarantine restrictions diminish.

Moreover, governments as well as peoples should ever remember that they have much more to dread from home-bred diseases never absent than from the occasional visitations of any foreign pestilence, however formidable it may be; and also that the very same measures which experience has shown will largely prevent the one will infallibly disarm the other of its power.

---

### Editorial Note.

It is fitting that we should add a note to the above well-timed article. We would direct particular attention to two "Parliamentary Papers" which have recently been published, the one on "Quarantine Practice;" the other on "Quarantine Laws." At the annual meeting of the National Association for the Promotion of Social Science, held in Liverpool in 1858, Dr. Milroy read a paper entitled "*Quarantine as it is, and as it ought to be.*" In consequence of a suggestion made in this paper, the Association passed a resolution to the effect that it was very desirable that a committee should be formed to investigate the subject of Quarantine in its various bearings.

This resolution was immediately carried into effect, and a committee was appointed, comprising several of the most influential members of the medical profession, to wit, Sir William Pym, Superintendent-General of Quarantine, the Directors-General of the Medical Departments of the Army and Navy, Sir James Clark, Sir Ranald Martin, Dr. Southwood Smith, &c., also three Members of Parliament representing Liverpool, Manchester, and Glasgow.

In carrying out the investigation, the committee framed a set of queries calculated to elicit information as to the actual existing condition, and the past results, of the practice of Quarantine in foreign countries, as well as in our own country and colonies. These queries were transmitted officially to all British consuls abroad, and to the governors of all British colonies, by order of the Foreign and Colonial Secretaries of State. Thus, the most authentic information from all parts of the world might reasonably be looked for, and a safe groundwork laid for a satisfactory examination of an intricate and hitherto unexplored subject.

The extent of the inquiry instituted may be gathered from the "Parliamentary Papers" already referred to, which have been drawn up by the honorary secretary of the committee, Dr. Milroy, and which contain a digest of the leading information transmitted to the committee from all quarters of the globe. Upon this information the report of the committee will be founded, and this, we understand, is now in course of preparation. We shall await the appearance of the document with considerable interest, but, in the meantime, we are wishful to call the attention of the profession to the consideration of a highly important subject of State Medicine, affecting the interest as well as the convenience of all maritime and commercial countries without exception, and one which it behoves all medical men to understand, more especially those attached to the public service, and all who are called upon to go abroad or to practise in our colonies. It is obvious, from the details contained in the two "Parliamentary Papers," that the utmost diversity of practice exists in different countries in reference to Quarantine, and that many vague and ignorant ideas prevail on the subject. It is, therefore, high time that it should be brought within the field of rational inquiry.

Besides the mass of information derived from the replies of British consuls and governors of colonies, and also of several medical officers of the army and navy, the first-dated "Parliamentary Paper" contains a summary of the proceedings of the International Conference on Quarantine, which was held in Paris in 1851-2, and at which two delegates—one consular and the other medical—attended from all the Southern States of Europe, and also from Great Britain and Russia, as having possessions in the Mediterranean and Black Sea. But little practical good resulted from their six-months' deliberations, in consequence, chiefly, it appears, from the disagreement of opinion among the medical members. No account of the proceedings of the Conference had been made public until the above summary was prepared from the original *procès verbaux* of the Conference, for the information of the Quarantine Committee of the Social Science Association.

In addition to the evidence respecting Quarantine contained in the two "Parliamentary Papers," they will be found to afford some very interesting information about the appearance of epidemic diseases in different years in various foreign countries and in our own colonies. Might not a useful hint be taken from this result to lay the foundation for a wide-spread registration of pestilential diseases from year to year, through the instrumentality of British consuls and colonial governors—a registration conducted somewhat after the plan adopted in respect of meteorological and magnetic inquiries, and addressed to one of the departments of the public service? For this notion we have primarily to thank Dr. Milroy, as, if we mistake not, it formed the basis of a scheme for the registration of epidemics, which he submitted to the Satistical Congress, held in London last year.

In concluding this note, it is rendering but meagre justice to Dr. Milroy to remark, that both the profession and the public will owe a deep debt of gratitude to him for his labours—self-imposed and self-sacrificing—on Quarantine.—Ed.

